# Dietary Supplementation with Trihexanoin Enhances Intestinal Function of Weaned Piglets

**DOI:** 10.3390/ijms19103277

**Published:** 2018-10-22

**Authors:** Tao Wu, Kang Li, Dan Yi, Lei Wang, Di Zhao, Yang Lv, Lin Zhang, Hongbo Chen, Binying Ding, Yongqing Hou, Guoyao Wu

**Affiliations:** 1 Engineering Research Center of Feed Protein Resources on Agricultural By-products, Ministry of Education, Hubei Key Laboratory of Animal Nutrition and Feed Science, Wuhan Polytechnic University, Wuhan 430023, China; wutao@whpu.edu.cn (T.W.); lee49127@163.com (K.L.); yidan810204@whpu.edu.cn (D.Y.); wanglei@whpu.edu.cn (L.W.); zhaodi@whpu.edu.cn (D.Z.); yanglyu.yl@gmail.com (Y.L.); chenhongbo@whpu.edu.cn (H.C.); dbying7471@126.com (B.D.); g-wu@tamu.edu (G.W.); 2 Yangtze River Fisheries Research Institute, Chinese Academy of Fishery Sciences, Wuhan 430223, China; zhanglhg@163.com; 3 Department of Animal Science, Texas A&M University, College Station, TX 77843, USA

**Keywords:** intestinal morphology and function, glycogen and fat metabolism, trihexanoin, weaned piglets

## Abstract

Trihexanoin is a short-chain triglyceride (SCT). Many studies have reported that SCTs play important roles in the maintenance of intestinal epithelial structure and function. The present work was to investigate the effects of trihexanoin on growth performance, carbohydrate and fat metabolism, as well as intestinal morphology and function in weaned piglets. Twenty weaned piglets (21 ± 2 d) were randomly allocated to one of two treatment groups: The control group (basal diet supplemented with 0.5% soya oil); the TH group (basal diet supplemented with 0.5% trihexanoin). Dietary trihexanoin supplementation significantly reduced diarrhea rate; increased the concentrations of LDL, HDL and total protein in plasma; decreased cholesterol concentrations and glutamyl transpeptidase activity in plasma; improved intestinal morphologic structure; altered the mRNA levels and abundances of proteins related to glycogen and fat metabolism, mucosal barrier function, antioxidant capacity and water transport capacity; and altered the community of intestinal microflora. These results indicate that dietary trihexanoin supplementation could reduce diarrhea, regulate carbohydrate and fat metabolism, exert beneficial effects on the intestinal mucosal barrier, protect the intestinal mucosa from injuries, improve intestinal transport and absorption, and enhance antioxidant capacity. In conclusion, dietary supplementation with 0.5% trihexanoin improves the intestinal function and health of weaned piglets.

## 1. Introduction

In recent years, the feeding stage of piglets has become one of the most important aspects in the swine industry [1]. Weaning of piglets occurs when food is changed from maternal milk to a solid diet, and this event impairs gut development and function because of many complex factors including environmental and dietary stresses [2]. Thus, weaning is one of the most critical developmental stages of the digestive tract [3]. The long-term occurrence of these symptoms caused by weaning could lead to high rates of morbidity and mortality of piglets [1]. This not only causes economic losses in pig production, but also increases public health risks due to the production of pathogenic bacteria-infected pork, which has troubled the global pig industry for many years [4].

Short-chain triglyceride (SCT), which is formed by short-chain fatty acids (SCFAs) and glycerol, plays an important role in maintaining intestinal morphological structure and function, which could be one way to alleviate the weaning problems [5]. SCT is readily absorbed by the intestine, and can serve as a metabolic fuel, reduce the osmotic pressure in the intestine, and boost the absorption of Na^+^ from the gut lumen [6]. Results of recent research indicated that SCFA can modulate intestinal pH, alleviate intestinal-mucosal injury under weaning stress [7], inhibit the proliferation of harmful bacteria, promote the growth of beneficial bacteria, and regulate immune responses [8]. In addition to food sources, SCFA is mainly produced by the microbial fermentation of non-digestible sugar in the colon and cecum [9].

There are several lines of evidence suggesting that short-term (7 to 10 days) intravenous administration of SCTs may be beneficial for cellular proliferation in the colon, thereby alleviating intestinal atrophy associated with trauma [10]. In rats with experimentally induced short-bowel syndrome, an elemental enteral diet containing SCTs increases intestinal adaptation as compared with a diet containing medium-chain triglycerides [11].

As a short-chain triglyceride (SCT), trihexanoin (TH, tricaproylglycerol) is water-soluble. When administered parenterally, trihexanoin is rapidly hydrolyzed to glycerol and hexanoic acid in the small intestine. However, it is still unknown whether or how trihexanoin could affect the intestinal function and whole-body growth of weaned piglets. The objective of this study was to determine the effects of dietary supplementation with trihexanoin on these variables in weaned piglets.

## 2. Results

### 2.1. Growth Performance

As shown in Table 1, there was no significant difference in average daily feed intake (ADFI), average daily gain (ADG), or the feed to gain ratio (F/G) between control and TH groups. However, dietary trihexanoin supplementation substantially decreased diarrhea rate (DR) during days 0 to 10 and days 0 to 21 of the study (*p* < 0.05).

### 2.2. Plasma Biochemical Indices

As shown in Table 2, compared with the control group, total protein (TP), total cholesterol (CHOL), blood urea nitrogen (BUN) and glutamyl transpeptidase (GGT) in plasma were obviously reduced in the TH group on day 10 (*p* < 0.05), whereas glucose (GLU) in plasma was significantly increased in the TH group on day 10 (*p* < 0.05); total protein (TP) was obviously increased in the TH group on day 20 (*p* < 0.05), and glutamyl transpeptidase (GGT) in plasma was significantly reduced in the TH group on day 20 (*p* < 0.05). There were no significant differences in other indices, including albumin (ALB), triglyceride (TG), alkaline phosphatase (ALP) and creatinine (Crea).

### 2.3. Concentrations of LDL and HDL in Plasma

**Figure 1:**
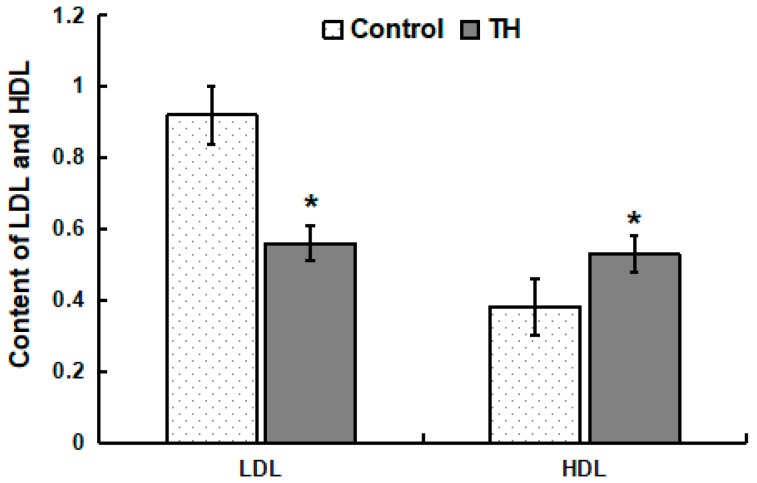
Content of LDL and HDL in plasma. Values are means ± SD, *n* = 6. * Means are significantly different from the control group (*p* < 0.05).

Within the control group, the concentration of HDL in plasma was lower (*p* < 0.05) than that of LDL (Figure 1). Within the TH group, the concentration of HDL in plasma was similar to that of LDL (Figure 1). Compared with the control group, the concentration of LDL in the plasma of the TH group decreased significantly while the concentration of HDL increased significantly (*p* < 0.05) (Figure 1).

### 2.4. Intestinal Morphology

**Figure 2:**
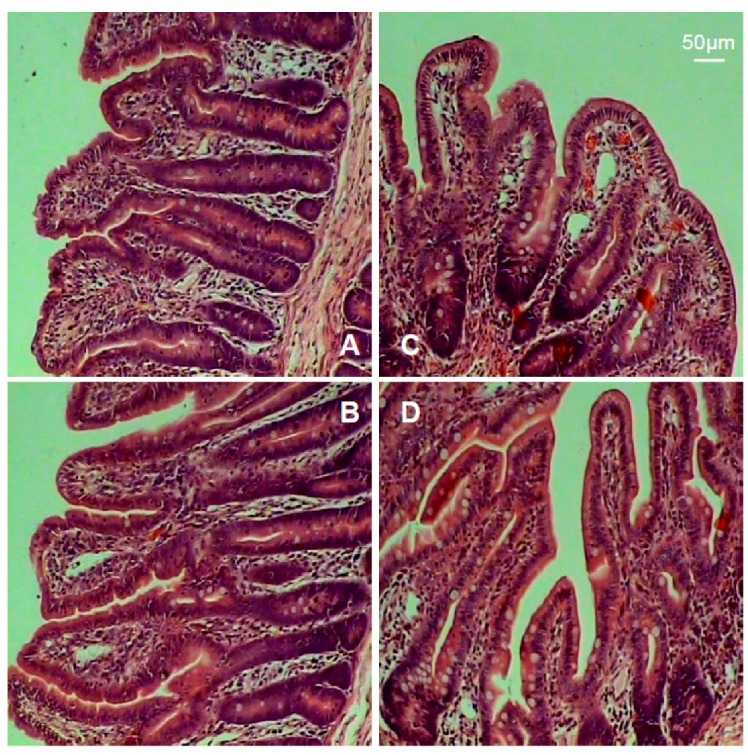
Intestinal mucosal morphology. (**A**) Jejunum (control). (**B**) Jejunum (TH group). (**C**) Ileum (control). (**D**) Ileum (TH group).

The intestinal morphology is shown in Figure 2, and the indexes including villus height and surface area, crypt depth, and the ratio of villus height to crypt depth are summarized in Table 3. Results showed that piglets in the TH group exhibited marked increases in villus height, surface area, and the ratio of villus height to crypt depth (*p* < 0.05).

### 2.5. Gene Expression

Compared with the control group, piglets in the TH group exhibited significant increases (*p* < 0.05) in the mRNA levels of *LIPE*, *LPL*, *PPARG*, *ACACA*, and *SLC27A2* in the intestine and mesenteric adipose tissue, of hepatic *ACACA*, as well as of jejunal *LPL* and *PPARG*; substantial reductions (*p* < 0.05) in the mRNA levels of *LIPE*, *LPL*, *FASN* and *SLC27A2* in liver, as well as *FASN* in the jejunum (Table 4).

Compared with the control group, dietary supplementation with trihexanoin increased (*p* < 0.05) the mRNA levels of *INSR* in the duodenum, jejunum and ileum, of *PCK1* and *AQP10* in four intestinal segments, of *AQP8* in the jejunum, ileum and colon, and of *Nrf2* in the jejunum and colon, while decreasing (*p* < 0.05) the mRNA levels of *ASS1* and *NOX2* in four intestinal segments as well as *Nrf2* and *GSTO2* in the duodenum (Table 5).

### 2.6. Protein Abundances

**Figure 3:**
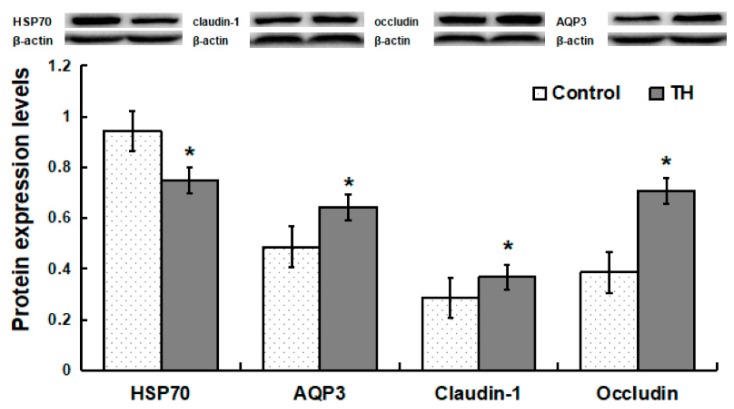
Protein expression levels in jejunum. Values are means ± SD, *n* = 6. * Means are significantly different from the control group (*p* < 0.05).

Compared with the control group, piglets in the TH group exhibited a decrease (*p* < 0.05) in the protein abundance of HSP70, but increases (*p* < 0.05) in the abundances of AQP3, claudin-1 and occludin proteins (Figure 3).

### 2.7. Intestinal Microflora

As was shown in Table 6, compared with the control group, pigs in the TH group exhibited decreases in the number of *Enterobacteriaceae* in the ileum (*p* < 0.05), and increases in the numbers of *Enterococcus*, *Clostridium*, *Lactobacillus* and *Bifidobacterium* in the ileum (*p* < 0.05); decreases in the numbers of *Enterobacteriaceae*, *Enterococcus* and *Lactobacillus* in the colon (*p* < 0.05); and decreases in the numbers of *Enterobacteriaceae*, *Clostridium*, *Lactobacillus* and *Bifidobacterium* in the cecum (*p* < 0.05). However, there was no significant difference in the total number of bacterium among the different groups of pigs.

## 3. Discussion

Piglet diarrhea is one of the most challenging problems in the pig industry, which causes a huge economic loss [12]. A major finding of this study is that diarrhea rate was markedly reduced by trihexanoin supplementation, indicating that trihexanoin could effectively relieve diarrhea in weaned piglets.

Protein is the important material basis of material metabolism, growth and development in animals. Most plasma proteins are synthesized by the liver, and the amount of total protein in blood reflects the metabolic activity of substances [13]. Blood cholesterol level is related to many cardiovascular diseases, and it is one of the blood routine testing indicators [14]. Cholesterol in blood is the main factor contributing to atherosclerosis, and both high and low levels of this lipid could impair animal health [15]. GGT is an essential enzyme in the metabolism of both proteins and amino acids, and it could reflect the injury of various cells by oxygen free radicals [16]. High levels of GGT in plasma may be an indicator of oxidative stress and its damage to the bile duct epithelium and liver [16]. Furthermore, the results of this research showed that the supplementation of trihexanoin could lower blood cholesterol, regulate the metabolism of proteins and amino acids, and decrease the stress-related reactions in the liver. Of note, although the concentrations of glucose and BUN in plasma showed significant differences between the treatment groups, their values were still within the normal range.

Insulin receptor (INSR) is a transmembrane receptor which belongs to the class of tyrosine kinase receptors and can be activated by insulin, IGF-I, and IGF-II [17]. Phosphoenolpyruvate carboxykinase 1 (PCK1) is a main control point for the regulation of gluconeogenesis, which can be regulated by insulin, glucocorticoids, glucagon, cAMP, and diet [18,19]. The protein encoded by the argininosuccinate synthase 1 (ASS1) gene catalyzes a step of intestinal arginine biosynthesis from glutamine, glutamate, proline, and aspartate [19]. A decreased activity of ASS1 may channel glutamate and aspartate toward pyrimidine synthesis in rapidly proliferating cells (e.g., enterocytes and tumors) by activating CAD (carbamoyl-phosphate synthase 2, aspartate transcarbamylase, and dihydroorotase complex) [20]. In this study, dietary supplementation with trihexanoin increased the expression of *INSR* and *PCK1* and decreased the expression of *ASS1* in bowels, suggesting that trihexanoin could substantially improve the capacity of hepatic glycogenesis and glycogenolysis, as well as positively regulate glycogen metabolism in weaned piglets. Measurements of whole-body glucose fluxes and hepatic glucose metabolism using isotopes are warranted to test this hypothesis. 

Intervention trials and prospective studies have shown that hypercholesterolemia, especially increases the concentrations of LDL cholesterol in plasma, leading to the development of atherosclerosis [21]. In contrast, one study demonstrated a negative correlation between plasma HDL cholesterol and cardiovascular disease [22]. In this study, dietary supplementation with trihexanoin positively regulated the concentrations of plasma LDL and HDL, indicating that trihexanoin could effectively reduce cholesterol and reduce fat deposition in pigs. 

The genes associated with fat metabolism in this study included hormone-sensitive lipase (*LIPE*), lipoprotein lipase (*LPL*), peroxisome proliferator-activated receptor gamma (*PPARG*), acetyl-CoA carboxylase 1 (*ACACA*), fatty acid synthase (*FASN*), and solute carrier family 27 member 2 (*SLC27A2*). *LIPE* plays vital roles in the digestion, transport and processing of dietary lipids [23]. *LPL* functions as a homodimer, and possesses the functions of both triglyceride hydrolase and ligand/bridging factor for receptor-mediated lipoprotein uptake [24]. *PPARG* regulates fatty acid storage and glucose metabolism, and has been implicated in the pathology of some diseases [25]. *ACACA* catalyzes the carboxylation of acetyl-CoA to malonyl-CoA, the rate-limiting step in fatty acid synthesis [26]. The main function of *FASN* is to catalyze the synthesis of palmitate from acetyl-CoA in the presence of NADPH, and thus, the formation of long-chain saturated fatty acids in the body [27]. *SLC27A2* converts free long-chain fatty acids into fatty acyl-CoA esters, plays a key role in lipid biosynthesis and fatty acid degradation [28]. In this study, dietary supplementation with trihexanoin altered the expression of genes related to fat metabolism, indicating that trihexanoin could promote fat synthesis in the liver but inhibit this biochemical process in the jejunum. Collectively, these results indicate that trihexanoin plays a crucial role in the regulation of fat metabolism in a tissue-specific manner.

Intestinal morphology indexes such as villus height, surface area, crypt depth, and the ratio of villus height to crypt depth are the common indicators of intestinal morphologic development and intestinal morphological integrity. Usually, the increases in villus height, villus surface area, as well as the ratio villus/crypt reflect the improvement of intestinal absorption capacity and intestinal health [29]. Intestinal epithelial integrity is maintained by cohesive interactions between cells via the formation of tight junctions. The members of the claudin-family play a critical role in tight junction formation and determine permeability characteristics in the gut [30]. Especially claudin-1 and occludin integrate diverse processes, such as gene transcription, tumor suppression, and cell proliferation [31]. Claudin-1 and occludin modulate intestinal-mucosal structure and function, and integrate diverse processes, such as gene transcription, tumor suppression, cell proliferation, and cell polarity [32]. Heat shock protein 70 (HSP70) protects cells from thermal or oxidative stress, and a high concentration of HSP70 is indicative of oxidative stress. The data of this study showed that dietary supplementation with trihexanoin improved intestinal growth and development, as indicated by (a) increases in intestinal villus height and surface area as well as the ratio of villus height to crypt depth, and the abundances of claudin-1 and occludin proteins; and (b) a reduction in the intestinal expression of HSP70. These results support the notion that trihexanoin could improve intestinal morphologic structure, maintain intestinal mucosal integrity, and exert beneficial effects on the epithelial barrier.

Aquaporins such as AQP3, AQP8 and AQP10 are important water channel proteins which conduct water into and out of the cell [33]. Nuclear factor like 2 (Nrf2) is a basic leucine zipper (bZIP) protein that regulates the expression of antioxidant proteins to protect the body against oxidative damage induced by injury and inflammation [34]. *NOX2* is a member of the NADPH oxidase family, which generates superoxide by transferring electrons from NADPH inside the cell across the membrane and coupling these processes to oxygen consumption to produce superoxide anion, a reactive free-radical [35]. Glutathione *S*-transferase omega-2 (GSTO2) exhibits glutathione-dependent thiol transferase activity, participates in the detoxification of inorganic arsenic, catalyzes the reduction of monomethylarsonic acid to monomethylarsonous acid, the rate limiting step in detoxification of inorganic arsenic [36]. Consistent with this view, we found that dietary supplementation with trihexanoin increased the mRNA levels of *AQP8, AQP10, Nrf2*, *NOX2* and *GSTO2*, as well as the abundances of the AQP4 protein. These results indicate that trihexanoin could substantially improve the capacity of the small intestine in water absorption, and antioxidant responses.

Animals coexist with different bacteria that protect the host from colonization of pathogenic bacteria, regulate intestinal growth and produce metabolites for use by the host [37]. The gradual conversion of piglets’ diets from liquid milk to solid diets will disturb the balance of intestinal microflora and have adverse effects on gastrointestinal function [38]. When the balance of the intestinal microflora is not maintained, excessive harmful bacterial metabolites will lead to diarrhea and various diseases in piglets [39]. In addition to having a direct impact on intestinal barrier function, SCFAs can also reduce the pH value of the gastrointestinal tract, which can promote the growth of probiotics, inhibit the growth of some intestinal bacterial pathogens, and may induce the secretion of some autolytic enzymes, leading to the death and dissolution of bacteria [40]. *Enterobacteriaceae* are divided into five groups: *Escherichia coli*, *Klebsiella*, *Proteus*, *Yersinia* and *Erwinia*, which are almost all pathogenic bacteria [41]. Although *Enterococcus* is thought to have an “active” role in cheese technology, its isolated populations have emerged as conditional pathogens for mammals [42]. *Clostridium* is a gram-positive bacterium, is anaerobic, aggregated after the formation of spores, and is an important cause of enteritis. *Bifidobacterium*, which is also a gram-positive anaerobic bacterium with its number decreasing with age in the intestine, is effective in treating diarrhea [43]. The results of this experiment showed that the content of *Enterobacter* in the ileum, colon and cecum decreased significantly after SCFA esters were added, and the number of *Clostridium* in the colon and cecum decreased significantly. These findings indicated that the addition of SCFA esters to diet had a good inhibitory effect on pathogenic bacteria in the intestine of piglets. This may also be one mechanism for trihexanoin to alleviate diarrhea.

## 4. Materials and Methods

### 4.1. Experimental Animals and Design

The animal use protocol for the present study was approved by the Animal Care and Use Committee at Wuhan Polytechnic University (2015-0316, 16 Mar 2015). Twenty crossbred healthy piglets (Duroc × Landrace × Yorkshire) were weaned at 21 days of age. After weaning, piglets had free access to the basal diet between 21 and 24 days of age (days 0–3 postweaning) for adapting to solid food. At 24 days of age, piglets (average body weight of 7.25 ± 1.13 kg) were assigned randomly into one of the two treatment groups: Control group (piglets fed the basal diet supplemented with 0.5% soya oil); TH group [piglets fed the basal diet supplemented with 0.5% trihexanoin (purity 97%; Sigma, CA, USA)]. The dose of trihexanoin was chosen according to results of a preliminary experiment, which showed that the piglets in the 0.5% trihexanoin group exhibited higher ADFI than that in the other groups (with 0%, 1% and 2% trihexanoin). 

Each piglet was individually housed in a 1.20 × 1.10 m^2^ steel metabolic cage with ten replicate cages per treatment. All diets were isocaloric. On days 10 and 20 of the trial, blood samples were collected from the anterior vena cava of piglets, and then all piglets were euthanized under anesthesia with an intravenous injection of pentobarbital sodium (50 mg/kg BW). Thereafter, the pig abdomen was opened immediately from sternum to pubis, the whole gastrointestinal tract was immediately exposed, and the liver was obtained for storage at −80 °C until assay. The small intestine was immediately dissected free of the mesentery on a chilled stainless-steel tray, and mesenteric adipose tissue samples were rapidly frozen in liquid nitrogen and stored at −80 °C until assay. Three intestinal segments (one 5-cm piece and two 10-cm pieces in length for each segment) were respectively cut at each distal duodenum, mid-jejunum and mid-ileum. The 5-cm segments were gently flushed with ice-cold PBS (phosphate buffered saline, pH 7.4) and placed in chilled formalin solution (10%), then processed by embedding and staining for the observation of intestinal morphology. The 10-cm segments were longitudinally cut and the contents were flushed with ice-cold PBS. Intestinal mucosa was scraped and rapidly frozen in liquid nitrogen, then stored at −80 °C until analysis. All samples were collected within 15 min.

### 4.2. Biochemical Indices, LDL and HDL in Plasma

Plasma biochemical indicators were measured with kits using a Hi-tachi 7060 Automatic Biochemical Analyzer (Hitachi, Tokyo, Japan), and low density lipoprotein (LDL) and high density lipoprotein (HDL) in plasma were analyzed using spectrophotometry with commercially available kits (Jiancheng Bioengineering Institute, Nanjing, China). Assays were performed in triplicate.

### 4.3. Intestinal Morphology

Intestinal tissue samples used for the morphometric study were dehydrated and embedded in paraffin, sectioned at a thickness of 4 mm, and stained with haematoxylin and eosin. Morphological measurements were carried out with a light microscope (Leica microsystems, Wetzlar, Germany) with the Leica Application Suite image analysis software (Leica microsystems, Wetzlar, Germany). Intestinal villus height and width, as well as crypt depth, were measured to calculate both the villus crypt ratio and villous surface area.

### 4.4. Quantitative PCR Analyses for Gene Expression and Intestinal Microflora

mRNA levels of genes in the liver and intestine samples were quantitated by the method of real-time quantitative PCR (qPCR). Approximately 100 mg of each frozen liver and intestinal sample was powdered, and total RNA was isolated with the use of the Trizol Reagent protocol (Invitrogen, Carlsbad, CA, USA). Total RNA was reverse-transcribed using a Prime Script^®^ RT reagent kit with gDNA Eraser (Takara, Dalian, China), and cDNA was synthesized and stored at −20 °C.

To amplify cDNA fragments, the primer pairs were designed and used for qPCR (Table 7). The qPCR was performed using the SYBR^®^ Premix Ex Taq^™^ (Takara, Shiga, Japan) with the 50 μL system on an Applied Biosystems 7500 Fast Real-Time PCR instrument (Foster City, CA, USA) [29,44]. *Ribosomal protein L4* (RPL4) and *glyceraldehyde-3-phosphate dehydrogenase* (GADPH) were used as the reference genes. Results were analyzed by the 2^−ΔCt^ method as described [29,44]. Each biological sample was run in triplicate.

The intestinal microflora was analyzed by the same method above, the primer pairs were also shown in Table 7, and the 16S RNA was used as the reference genes.

### 4.5. Protein Immunoblot Analysis

The abundances of proteins were analyzed by using the Western blotting technique. The samples (0.1 mL) were homogenized in 1 mL of lysis buffer using a Polytron homogenizer and centrifuged at 12,000 × *g* for 15 min at 4 °C. The supernatant fluid was aliquoted into micro-centrifuge tubes, to which 2 × SDS sample buffer (2 mL of 0.5 mol/L Tris, pH 6.8, 2 mL glycerol, 2 mL of 10% SDS, 0.2 mL of β-mercaptoethanol, 0.4 mL of a 4% solution of bromophenol blue, and 1.4 mL of water) was added in a 1:1 ratio. The samples were boiled and cooled on ice before use for western blotting. Proteins were separated by electrophoresis on a 10% polyacrylamide gel, and then electrophoretically transferred to a polyvinylidene difluoride (PVDF) membrane. Skim-milk powder in TBST buffer (1×Tris-buffered saline including 0.1% Tween 20) was used to blot the membrane for 1 h at 25 °C. Membranes were incubated overnight at 4 °C with one of the primary antibodies: AQP3 and AQP4 (rabbit, 1:1000; Cell Signaling Technology, Danvers, MA, USA), occludin and villin (mouse, 1:1000; Sant Cruze Biotechnology, CA, USA), HSP70 and claudin-1 (mouse, 1:1000; Invitrogen, Carlsbad, CA, USA), β-actin (mouse, 1:2000; Sigma, St. Louis, MO, USA). Thereafter, the membranes were washed with TBS-T and incubated for 1 h at 25 °C with the secondary antibody: an anti-rabbit (1:2000; Invitrogen, Carlsbad, CA, USA) or anti-mouse (1:2000; Invitrogen, Carlsbad, CA, USA) antibody. After being washed with TBST, blots on the membrane were developed with the use of an Enhanced Chemiluminescence Western blotting kit (Amersham Biosciences, Little Chalfont, UK), visualized, and quantified using an imaging system (Alpha Innotech, San Leandro, CA, USA). Abundances of all proteins of interest were normalized to those for β-actin.

### 4.6. Statistical Analysis

All data are expressed as means ± SD. Differences between means were determined by the Student’s unpaired *t*-test. The data were analyzed by using the SPSS13.0 software (SPSS Inc., Chicago, IL, USA). Probability values ≤ 0.05 was considered statistically significant.

## 5. Conclusions

Dietary supplementation with 0.5% trihexanoin effectively reduced diarrhea incidence, decreased stress-related reactions in the liver, and altered the community of intestinal microflora. Trihexanoin supplementation also regulated glycogen and fat metabolism in the intestine, adipose tissue and liver, while improving intestinal morphologic structure, mucosal barrier function, antioxidant capacity and water transport capacity in weaned piglets. These findings have important implications for the improvement of nutrition and intestinal health in young pigs and other mammals.

## Figures and Tables

**Table 1:** The growth performance of piglets receiving dietary supplementation with or without trihexanoin (TH).

Item	Day 0 to Day 10		Day 11 to Day 21		Day 0 to Day 21	
	Control	TH	Control	TH	Control	TH
ADG/g	244.5 ± 52.0	267.8 ± 91.5	437.8 ± 69.8	475.5 ± 99.3	341.1 ± 56.0	371.63 ± 86.07
ADFI/g	308.1 ± 8.3	339.5 ± 126.1	662.3 ± 86.1	739.4 ± 139.9	485.2 ± 44.1	539.47 ± 119.3
F/G	1.28 ± 0.19	1.26 ± 0.07	1.52 ± 0.10	1.56 ± 0.05	1.43 ± 0.09	1.45 ± 0.04
DR/%	15.00 ± 10.69 ^b^	1.25 ± 3.54 ^a^	2.50 ± 4.63	1.25 ± 3.54	8.33 ± 6.61 ^b^	1.19 ± 2.20 ^a^

Values are means ± SD, *n* = 6. ^a, b^ Means within rows with different superscripts differ (*p* < 0.05).

**Table 2:** Biochemical indices in the plasma of piglets receiving dietary supplementation with or without trihexanoin (TH).

Item	Sampling on Day 10		Sampling on Day 20	
	Control	TH	Control	TH
TP (g/L)	51.05 ± 2.94 ^a^	52.28 ± 2.32 ^b^	51.79 ± 2.44 ^a^	53.06 ± 2.05 ^b^
ALB (g/L)	30.26 ± 2.45	31.16 ± 2.21	30.16 ± 1.77	30.81 ± 1.51
CHOL (mmol/L)	1.82 ± 0.26 ^b^	1.62 ± 0.12 ^a^	1.83 ± 0.10	1.94 ± 0.20
TG (mmol/L)	0.40 ± 0.10	0.37 ± 0.08	0.48 ± 0.10	0.53 ± 0.09
ALP (U/L)	341.76 ± 57.49	316.15 ± 31.38	314.71 ± 59.44	302.25 ± 37.29
BUN (mmol/L)	3.10 ± 1.04 ^b^	2.67 ± 0.44 ^a^	2.91 ± 0.96	3.36 ± 0.76
Creatine (µmol/L)	81.88 ± 6.20	86.50 ± 7.25	78.00 ± 6.28	84.38 ± 8.72
Glucose (mmol/L)	5.32 ± 0.44 ^a^	5.94 ± 1.39 ^b^	5.54 ± 0.64	5.38 ± 0.48
GGT (mmol/L)	35.05 ± 5.21 ^b^	30.96 ± 5.75 ^a^	30.44 ± 4.00 ^b^	28.44 ± 2.80 ^a^

Values are means ± SD, *n* = 6. ^a, b^ On Day 10 or 20, means within a row with different superscript letters differ (*p* < 0.05).

**Table 3:** Intestinal morphology indexes of piglets receiving dietary supplementation with or without trihexanoin (TH).

Item	Jejunum		Ileum	
	Control	TH	Control	TH
Villus height (μm)	262.3 ± 12.5 ^a^	308.8 ± 19.1 ^b^	259.6 ± 23.5 ^a^	297.5 ± 21.1 ^b^
Crypt depth (μm)	60.35 ± 3.72	63.29 ± 6.43	66.01 ± 9.92	66.90 ± 13.15
Ratio of villus height to crypt depth	4.35 ± 0.23 ^a^	4.92 ± 0.47 ^b^	3.97 ± 0.33 ^a^	4.41 ± 0.12 ^b^
Villus surface area (μm^2^)	24,553 ± 3018 ^a^	36,786 ± 1725 ^b^	22,616 ± 3254 ^a^	32,462 ± 8389 ^b^

Values are means ± SD, *n* = 6. ^a, b^. For each segment of the small intestine, means within a row with different superscripts differ (*p* < 0.05).

**Table 4:** Relative expression levels of genes associated with fat metabolism in tissues of piglets receiving dietary supplementation with or without trihexanoin (TH).

Item	Fat		Liver		Jejunum	
	Control	TH	Control	TH	Control	TH
*LIPE*	1.00 ± 0.17 ^a^	1.61 ± 0.35 ^b^	1.00 ± 0.23 ^b^	0.81 ± 0.13 ^a^	1.00 ± 0.22	0.99 ± 0.07
*LPL*	1.00 ± 0.27 ^a^	1.73 ± 0.44 ^b^	1.00 ± 0.19 ^b^	0.73 ± 0.19 ^a^	1.00 ± 0.20 ^a^	1.55 ± 0.28 ^b^
*PPARG*	1.00 ± 0.24 ^a^	1.63 ± 0.39 ^b^	1.00 ± 0.19	1.10 ± 0.21	1.00 ± 0.24 ^a^	1.34 ± 0.25 ^b^
*ACACA*	1.00 ± 0.22 ^a^	1.90 ± 0.36 ^b^	1.00 ± 0.10 ^a^	1.26 ± 0.21 ^b^	1.00 ± 0.15	1.09 ± 0.12
*FASN*	1.00 ± 0.19	1.06 ± 0.27	1.00 ± 0.26 ^b^	0.46 ± 0.09 ^a^	1.00 ± 0.23 ^b^	0.49 ± 0.12 ^a^
*SLC27A2*	1.00 ± 0.27 ^a^	2.04 ± 0.42 ^b^	1.00 ± 0.23 ^b^	0.72 ± 0.12 ^a^	1.00 ± 0.22	0.99 ± 0.07

Values are means ± SD, *n* = 6. ^a, b^. For each tissue, means within rows with different superscripts differ (*p* < 0.05).

**Table 5:** Relative expression levels of genes associated with intestinal glycogen metabolism and function in tissues of piglets receiving dietary supplementation with or without trihexanoin (TH).

Item	Duodenum		Jejunum		Ileum		Colon	
	Control	TH	Control	TH	Control	TH	Control	TH
*INSR*	1.00 ± 0.21 ^a^	1.40 ± 0.24 ^b^	1.00 ± 0.17 ^a^	1.55 ± 0.36 ^b^	1.00 ± 0.19 ^a^	1.65 ± 0.28 ^b^	1.00 ± 0.22	0.99 ± 0.19
*PCK1*	1.00 ± 0.23 ^a^	2.06 ± 0.43 ^b^	1.00 ± 0.16 ^a^	2.29 ± 0.47 ^b^	1.00 ± 0.22 ^a^	2.02 ± 0.39 ^b^	1.00 ± 0.23 ^a^	2.42 ± 0.42 ^b^
*ASS1*	1.00 ± 0.27 ^b^	0.40 ± 0.11 ^a^	1.00 ± 0.18 ^b^	0.55 ± 0.13 ^a^	1.00 ± 0.26 ^b^	0.59 ± 0.13 ^a^	1.00 ± 0.26 ^b^	0.69 ± 0.16 ^a^
*AQP8*	1.00 ± 0.25	1.05 ± 0.23	1.00 ± 0.22 ^a^	1.45 ± 0.37 ^b^	1.00 ± 0.24 ^a^	2.20 ± 0.45 ^b^	1.00 ± 0.26 ^a^	2.30 ± 0.51 ^b^
*AQP10*	1.00 ± 0.17 ^a^	2.35 ± 0.51 ^c^	1.00 ± 0.22 ^a^	1.92 ± 0.44 ^c^	1.00 ± 0.21	1.61 ± 0.36 ^b^	1.00 ± 0.24	1.52 ± 0.33 ^b^
*Nrf2*	1.00 ± 0.20	0.75 ± 0.12 ^a^	1.00 ± 0.23 ^a^	1.59 ± 0.41 ^b^	1.00 ± 0.13 ^b^	1.10 ± 0.25 ^b^	1.00 ± 0.23 ^a^	1.42 ± 0.37 ^b^
*NOX2*	1.00 ± 0.20 ^b^	0.73 ± 0.16 ^a^	1.00 ± 0.22 ^b^	0.70 ± 0.12 ^a^	1.00 ± 0.14 ^b^	0.79 ± 0.10 ^a^	1.00 ± 0.13 ^b^	0.88 ± 0.15 ^a^
*GSTO2*	1.00 ± 0.23 ^a^	0.87 ± 0.21 ^a^	1.00 ± 0.23 ^a^	1.01 ± 0.11 ^a^	1.00 ± 0.19 ^a^	1.01 ± 0.11 ^a^	1.00 ± 0.25 ^a^	0.95 ± 0.23 ^a^

Values are means ± SD, *n* = 6. ^a, b, c^. For each tissue, means within a row with different superscripts differ (*p* < 0.05).

**Table 6:** Intestinal microflora of piglets.

Item	Ileum		Colon		Cecum	
	Control	TH	Control	TH	Control	TH
Total bacterium	1.00 ± 0.29	1.02 ± 0.26	1.00 ± 0.13	0.99 ± 0.13	1.00 ± 0.04	0.98 ± 0.05
*Enterobacteriaceae*	1.00 ± 0.17 ^b^	0.24 ± 0.05 ^a^	1.00 ± 0.19 ^b^	0.81 ± 0.22 ^a^	1.00 ± 0.17 ^b^	0.49 ± 0.07 ^a^
*Enterococcus*	1.00 ± 0.12 ^a^	2.17 ± 0.26 ^b^	1.00 ± 0.28 ^b^	0.35 ± 0.08 ^a^	1.00 ± 0.18	0.77 ± 0.14
*Clostridium*	1.00 ± 0.23 ^a^	3.28 ± 0.31 ^b^	1.00 ± 0.19	0.94 ± 0.27	1.00 ± 0.25 ^b^	0.68 ± 0.18 ^a^
*Lactobacillus*	1.00 ± 0.09 ^a^	3.93 ± 0.71 ^b^	1.00 ± 0.23 ^b^	0.61 ± 0.12 ^a^	1.00 ± 0.15 ^b^	0.31 ± 0.26 ^a^
*Bifidobacterium*	1.00 ± 0.15 ^a^	3.40 ± 0.55 ^b^	1.00 ± 0.26	1.08 ± 0.22	1.00 ± 0.24 ^b^	0.64 ± 0.15 ^a^

Values are means ± SD, *n* = 6. ^a, b^. For each segment of the intestine, means within a row with different superscripts differ (*p* < 0.05).

**Table 7:** Sequences of the primers used for quantitative RT-PCR analysis.

Genes	Forward Sequences	Reverse Sequences
*AQP8*	TGTGTCTGGAGCCTGCATGAAT	AGCAGGAATCCCACCATCTCA
*AQP10*	TGTCTGCTTTCTGTGCCTCTG	GGATGCCATTGCTCAAGGATAGATAA
*Nrf2*	GAAGTGATCCCCTGATGTTGC	ATGCCTTCTCTTTCCCCTATTTCT
*NOX2*	TGTATCTGTGTGAGAGGCTGGTG	CGGGACGCTTGACGAAA
*GSTO2*	GCCTTGAGATGTGGGAGAGAA	AAGATGGTGTTCTGATAGCCAAGA
*INSR*	GGGGCTAAAGAGGAACTATGAGG	AGAGGAAAGCGAAGACAGGAAA
*PCK1*	CGGGATTTCGTGGAGA	CCTCTTGATGACACCCTCT
*ASS1*	CCCTCACTTTGCCCATCTCT	CCCTACCCTTCCGTTTGCT
*LIPE*	CCAGCCCTGCCTTAATGTG	TCCCGAATACCCGCAAAG
*PPARG*	AGGACTACCAAAGTGCCATCAAA	GAGGCTTTATCCCCACAGACAC
*ACACA*	TGGCAGTGGTCTTCGTGTG	TCATCCACATCCTTCACATAACCT
*FASN*	ACACCTTCGTGCTGGCCTAC	ATGTCGGTGAACTGCTGCAC
*LPL*	AGCCTGAGTTGGACCCATGT	CTCTGTTTTCCCTTCCTCTCTCC
*SLC27A2*	TTTTCAGCCAGCCACTTTTG	CATTTGGTTTCTGGGGAGAGTT
*RPL4*	GAGAAACCGTCGCCGAAT	GCCCACCAGGAGCAAGTT
*GADPH*	CGTCCCTGAGAGACACGATGGT	GCCTTGACTGYGCCGTGGAAT
Total bacterium	CGGYCCAGACTCCTACGGG	TTACCGCGGCTGCTGGCAC
*Enterobacteriaceae*	CATTGACGTTACCCGCAGAAGAAGC	CTCTACGAGACTCAAGCTTGC
*Enterococcus*	CCCTTATTGTTAGTTGCCATCATT	ACTCGTTGTACTTCCCATTGT
*Clostridium*	AATGACGGTACCTGACTAA	CTTTGAGTTTCATTCTTGCGAA
*Lactobacillus*	AGCAGTAGGGAATCTTCCA	CACCGCTACACATGGAG
*Bifidobacterium*	GATTCTGGCTCAGGATGAACG	CGGGTGCTCCCACTTTCATG
*16S RNA*	CAGAAATGGGAATGGAAAGTTG	CCATTGGTCAGGTCATTCAATACA
